# World Health Organization Early Warning, Alert and Response System in the Rohingya Crisis, Bangladesh, 2017–2018

**DOI:** 10.3201/eid2411.181237

**Published:** 2018-11

**Authors:** Basel Karo, Christopher Haskew, Ali S. Khan, Jonathan A. Polonsky, Md Khadimul Anam Mazhar, Nilesh Buddha

**Affiliations:** Public Health England, London, UK (B. Karo);; European Centre for Disease Prevention and Control, EPIET European Programme for Intervention Epidemiology Training, Stockholm, Sweden (B. Karo);; World Health Organization, Geneva, Switzerland (C. Haskew, J.A. Polonsky);; University of Nebraska Medical Center, Omaha, Nebraska, USA (A.S. Khan);; World Health Organization, Cox's Bazar, Bangladesh (M.K.A. Mazhar);; World Health Organization Regional Office for South-East Asia, New Delhi, India (N. Buddha)

**Keywords:** Rohingya, Rohingya crisis, emergency response, EWARS, mobile phone–based surveillance, humanitarian innovation, outbreak detection, Bangladesh, surveillance

## Abstract

The Early Warning, Alert and Response System (EWARS) is a web-based system and mobile application for outbreak detection and response in emergency settings. EWARS provided timely information on epidemic-potential diseases among >700,000 Rohingya refugees across settlements. EWARS helped in targeting new measles vaccination campaigns and investigating suspected outbreaks of acute jaundice syndrome.

The international humanitarian system faces unprecedented challenges with the number of persons displaced by natural disasters and escalating conflicts at its highest in decades (*1*). Understanding the needs of crisis-affected persons and orchestrating rapid response play decisive factors in the effectiveness of humanitarian aid. Innovative technology and products can enhance the provision and quality of humanitarian assistance to contend with these growing challenges (*2*,*3*).

Since August 2017, violence in Myanmar’s Rakhine State has driven hundreds of thousands of Rohingya persons across the border into refugee settlements in Cox’s Bazar, Bangladesh (*4*,*5*). The poor environmental conditions and extremely high population density coupled with a preexisting lack of health services have left the Rohingya community vulnerable to communicable diseases and outbreaks. As a part of a massive response, the World Health Organization (WHO), in partnership with the Bangladesh Ministry of Health and Family Welfare (MoHFW), has implemented an Early Warning, Alert and Response System (EWARS) across the Rohingya settlements. EWARS is a web-based system and mobile application designed to enhance disease surveillance and outbreak detection in emergency settings (*6*,*7*). EWARS includes an analytic and alert module that signals outbreak at early stages and incorporates a risk assessment framework and matrix. EWARS can be deployed easily; it comes with all the equipment needed to establish surveillance and response activities, including 60 mobile phones, tablets, a local server, and a solar generator and solar chargers (Figure 1). A single kit costs approximately US $15,000 and can support surveillance in up to 60 fixed or mobile clinics serving ≈500,000 persons. WHO developed EWARS in 2015 and has deployed it in humanitarian crises, disease outbreaks, and natural disasters in South Sudan, Chad, Nigeria, Fiji, and Yemen (*6*,*8*,*9*).

**Figure 1:**
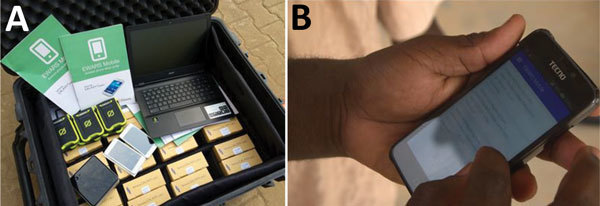
Examples of the World Health Organization EWARS supplies used for public health surveillance of Rohingya refugee populations in Bangladesh, 2017–2018. A) EWARS in a box. B) EWARS mobile application. EWARS, Early Warning, Alert and Response System.

In December 2017, WHO sent 2 “EWARS in box” kits (Figure 1, panel A) to the WHO office in Cox’s Bazar. Over 2 weeks, the WHO team organized 2 workshops and a series of field visits, in which the staff of 151 health facilities run by 23 humanitarian organizations were enrolled and trained as reporting sites for EWARS across the refugee settlements, serving >700,000 Rohingya refugees. However, the team continued supportive supervision visits for EWARS upon request, particularly when a new partner or health facility started operating in the camp. WHO supported facilities operating in remote field settings without reliable Internet or electricity by providing mobile phones, a local server, and solar chargers.

The case management team, composed of staff from WHO, MoHFW, and humanitarian organizations, defined the reportable diseases and their alert thresholds on the basis of burden and epidemic potential. The diseases included were acute watery diarrhea, bloody diarrhea, acute respiratory infection, measles/rubella, acute flaccid paralysis, suspected meningitis, acute jaundice syndrome, suspected hemorrhagic fever, neonatal tetanus, adult tetanus, malaria, unexplained fever, and severe malnutrition. Identified diseases were encoded in EWARS as a weekly report of new cases aggregated by site, age, and sex. By the first week of January 2018, humanitarian organizations submitted data by the EWARS mobile application, and the data were immediately available in the EWARS web application for analysis. The WHO team reviewed and managed all alerts triggered by EWARS on a weekly basis, following the workflow: alert verification, risk assessment, risk characterization, and outcome (discard, monitor, or respond). On average, there were ≈100 alerts per week; most were discarded as false alerts, because of data entry mistakes or because they did not meet the case definition or no cluster was identified. EWARS generated automated weekly bulletins that included reporting performance, trend and location of reported diseases, and summary of alerts triggered. The weekly bulletins were disseminated among partners and posted on WHO and MoHFW websites.

Information obtained by the weekly bulletins played an important role in driving public health action. For example, mapping alerts related to measles in EWARS and identifying the affected age groups helped in targeting new vaccination campaigns (Figure 2, panel A). In addition, alerts triggered by EWARS detected clusters of acute jaundice syndrome cases (Figure 2, panel B). Within 1 week, all health facilities were informed of the clusters (with age and site of the case-patients) and a team from the Bangladeshi Institute of Epidemiology, Disease Control and Research (IEDCR) was sent to the field to verify the cluster and collect samples for testing and identifying the etiology of the outbreak. This practice resulted in prompt detection of an outbreak of hepatitis A and the early initiation of appropriate intervention.

**Figure 2:**
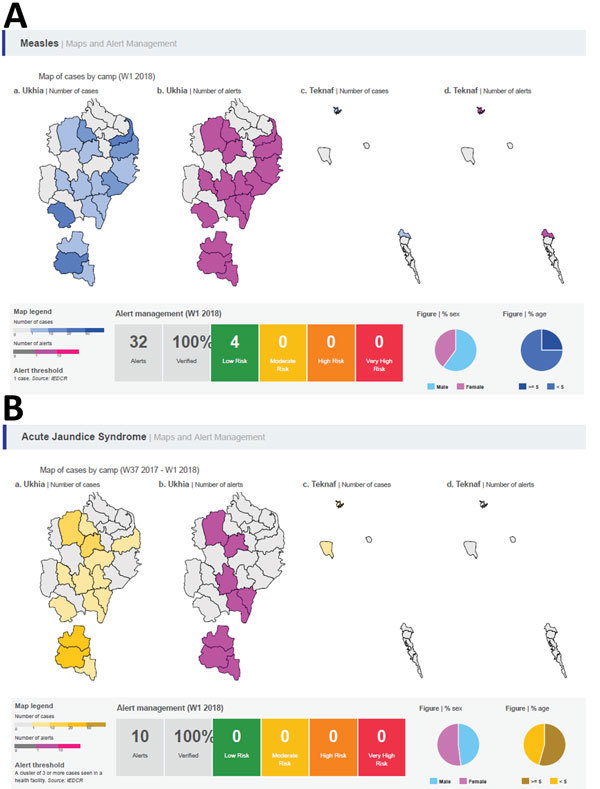
Screenshots of maps and alerts generated by the World Health Organization Early Warning, Alert and Response System during week 1, January 2018, in Rohingya refugee settlements in Cox’s Bazar, Bangladesh. A) Measles; B) acute jaundice syndrome.

EWARS was also used for daily monitoring of a diphtheria outbreak (reporting form, contact tracing form, treatment centers bed counts, and diphtheria antitoxin use). Previously, these forms were submitted daily as spreadsheets; the submitted case reports were noteworthy for being incomplete and with inconsistent variable names and data. Despite challenges in connectivity and data sharing, these new real-time records permitted a more accurate assessment of final outcomes for cases and postdischarge complications. EWARS also enabled the treatment centers to update incomplete data provided during the height of the outbreak from abstracting paper medical records.

A key limitation of the application itself was the lack of availability of mobile networks in some areas of the Rohingya settlements, which was a challenge for partners when trying to edit and update case-based records during the diphtheria outbreak. Although the mobile application can be used offline, the need for case-based records to be regularly edited made a laptop more convenient for this activity. A key lesson learned, therefore, was the need to have a fully offline version of the EWARS application with the capability for installation and use on laptop computers without any Internet connection, before being synchronized with a central server when connection allows.

EWARS is a successful example of innovative technology in humanitarian response with a positive impact for its users and crisis-affected communities. It provides a functional and simple digital surveillance system that can be easily deployed (“EWARS in a box”) and rapidly implemented in an emergency setting for timely detection of and response to new outbreaks. It has succeeded in disease monitoring, detecting outbreaks, and driving public health action among the Rohingya refugee population in Cox’s Bazar.
